# Associations of education and work status with alcohol use and cessation among pregnant women in Japan: the Tohoku Medical Megabank Project Birth and Three-Generation Cohort Study

**DOI:** 10.1186/s12889-021-11461-w

**Published:** 2021-07-15

**Authors:** Keiko Murakami, Taku Obara, Mami Ishikuro, Fumihiko Ueno, Aoi Noda, Shinichi Kuriyama

**Affiliations:** 1 Tohoku Medical Megabank Organization, Tohoku University, 2-1 Seiryo-machi, Aoba-ku, Sendai, Miyagi 980-8573 Japan; 2 Graduate School of Medicine, Tohoku University, 2-1 Seiryo-machi, Aoba-ku, Sendai, Miyagi 980-8575 Japan; 3 Department of Pharmaceutical Sciences, Tohoku University Hospital, 1-1 Seiryo-machi, Aoba-ku, Sendai, Miyagi 980-8574 Japan; 4 Department of Disaster Public Health, International Research Institute of Disaster Science, Tohoku University, 2-1 Seiryo-machi, Aoba-ku, Sendai, Miyagi 980-8573 Japan

**Keywords:** Alcohol cessation, Alcohol use, Early pregnancy, Japan, Middle pregnancy, Pregnant women, Prospective cohort study

## Abstract

**Background:**

There is inconsistent evidence on the associations of education and work status with alcohol use during pregnancy. Our aim was to examine the associations of education and work status with alcohol use and alcohol cessation during pregnancy in Japan.

**Methods:**

Data were analyzed from 11,839 pregnant women who participated in the Tohoku Medical Megabank Project Birth and Three-Generation Cohort Study from 2013 to 2017 in Japan. Women were dichotomized as current drinkers or non-drinkers in both early and middle pregnancy. Alcohol cessation was defined as alcohol use in early pregnancy, but not in middle pregnancy. Multivariable log-binomial regression analyses were conducted to examine associations of education and work status with alcohol use in early and middle pregnancy and alcohol cessation, adjusted for age and income. The prevalence ratios (PRs) and 95% confidence intervals (CIs) were calculated by work status and education.

**Results:**

The prevalence of alcohol use in early and middle pregnancy was 20.9 and 6.4%, respectively. Higher education was associated with alcohol use in early pregnancy both among working and non-working women; the PRs of university education or higher compared with high school education or lower were 1.62 (95% CI, 1.34–1.96) and 1.29 (95% CI, 1.16–1.45), respectively. Higher education was associated with alcohol cessation during pregnancy among working women; the corresponding PR was 1.09 (95% CI, 1.01–1.17). Working was associated with alcohol use in early and middle pregnancy. Working was associated with a decreased probability of alcohol cessation among women with lower education but with an increased probability of alcohol cessation among women with higher education; the PRs of working compared with not working were 0.91 (95% CI, 0.82–1.00) and 1.10 (95% CI, 1.00–1.20), respectively.

**Conclusions:**

Women with higher education were more likely to consume alcohol in early pregnancy and to cease alcohol use between early and middle pregnancy, especially working women. Working women were more likely to consume alcohol throughout pregnancy. Working women with lower education were less likely to cease alcohol use, whereas working women with higher education were more likely to cease alcohol use between early and middle pregnancy.

**Supplementary Information:**

The online version contains supplementary material available at 10.1186/s12889-021-11461-w.

## Background

Alcohol can readily cross the placenta, which results in damage to the organs of the embryo and fetus. Alcohol use during pregnancy can cause pregnancy complications such as low birth weight, preterm birth, and small for gestational age [1, 2], and can result in a range of lifelong disabilities known as fetal alcohol spectrum disorders [2, 3]. Because no amount of alcohol and no time to drink can be considered safe during pregnancy [2, 4], recommendations for alcohol use during pregnancy advocate abstinence in most countries [5]. Despite such recommendations, one meta-analysis published in 2017 estimated that the global prevalence of alcohol use during pregnancy was 9.8% [6].

Identification of pregnant women who are most likely to consume alcohol is essential for targeted interventions. Although a wide range of social factors have been examined as potential predictors, there is inconsistent evidence on the associations of education and work status with alcohol use during pregnancy. One systematic review showed that the associations of education were positive in two studies, negative in one, and null in eight, whereas the associations of work status were positive in two studies, negative in none, and null in five [7]. There are two possible explanations for these inconsistencies. One is that different studies have assessed alcohol use at different time points. Most studies examining alcohol use have been conducted using postpartum retrospective reports or at only one time point during pregnancy. Retrospective reports on postpartum women are subject to imprecise estimates of when they ceased alcohol use during pregnancy [8]. The prevalence of alcohol use is reportedly different in early pregnancy from middle/late pregnancy [9–14]. Although this suggests that the associations of education and work status with alcohol use may differ by pregnancy stage, very few studies have been conducted at different points during pregnancy among women who had already become aware of their pregnancy [15–17].

The other possible explanation is that social and cultural contexts influence varying social patterns of alcohol use in different countries [18]. Studies in Japan are needed because Japanese women have unique characteristics of alcohol use with respect to education and work status. In terms of education, there are many opportunities to inform pregnant women of the health risks of alcohol use from very early pregnancy, including the distribution of maternal and child health handbooks as soon as pregnancy is confirmed and the provision of health checkups for all pregnant women [19, 20]. Only limited evidence in Japan showed that highly educated women were less likely to consume alcohol after becoming aware of their pregnancy [9, 21]. In terms of work status, drinking with work colleagues is customary because alcohol use is often an integral part of social life [22, 23]. One national survey regarding alcohol use among Japanese women showed that working women had a higher risk of harmful alcohol use than non-working women [23]. It is therefore possible that these characteristics can affect the associations of education and work status with alcohol use among pregnant women in Japan.

Therefore, our aim was to examine the associations of education and work status with alcohol use in early and middle pregnancy and alcohol cessation between early and middle pregnancy in Japan.

## Methods

### Study population

**Fig. 1:**
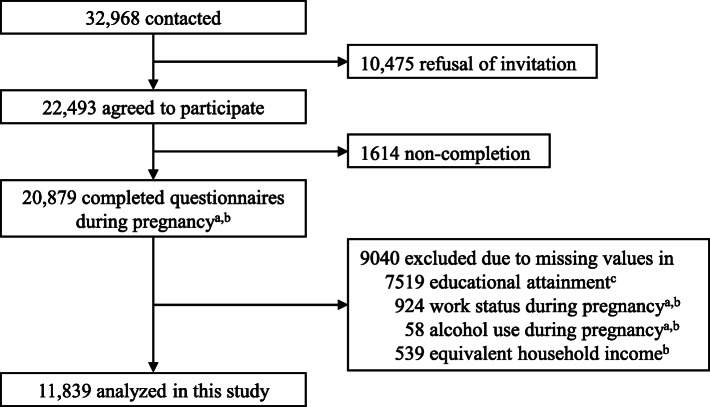
Flow diagram of participants in the present analysis of the TMM BirThree Cohort Study. ^a^Data on work status and alcohol use were obtained using a questionnaire administered in early pregnancy. ^b^Data on work status, alcohol use, and equivalent household income were obtained using a questionnaire administered in middle pregnancy. ^c^Data on educational attainment were obtained using a questionnaire administered 1 year postpartum

We used data obtained from the Tohoku Medical Megabank Project Birth and Three-Generation Cohort Study (TMM BirThree Cohort Study), details of which has been described elsewhere [24]. The aim of this cohort study was to evaluate the complex interactions of genetic and environmental factors using information on in utero and subsequent pediatric exposures and to assess maternal, pediatric, and family outcomes by evaluating a birth cohort and members of three generations [24]. Pregnant women and their family members were contacted in obstetric clinics or hospitals in Miyagi Prefecture when they scheduled their deliveries from 2013 to 2017. Tohoku University Tohoku Medical Megabank Organization established seven community support centers in Miyagi Prefecture for voluntary admission-type recruitment and health assessment of the participants [25]. Genome medical research coordinators in each clinic, hospital, or community support center provided information on the TMM BirThree Cohort Study to potential participants and to receive signed informed consent forms from those who agreed to participate. Of 32,968 pregnant women who were contacted, 22,493 agreed to participate and 20,879 completed the questionnaires in early pregnancy (< 14 weeks of gestation) and middle pregnancy (14–27 weeks of gestation). Among them, 9040 women were excluded because of missing values in educational attainment, work status, alcohol use, or equivalent household income. The remaining 11,839 pregnant women were included in the present study. Figure 1 shows the flow diagram of the present study. The TMM BirThree Cohort Study protocol was reviewed and approved by the Ethics Committee of Tohoku University Tohoku Medical Megabank Organization (2013-1-103-1). The characteristics of 11,839 analyzed women and 9040 excluded women are shown in Supplementary Table 1.

### Measures

Educational attainment was categorized into three groups: high school or lower (elementary, junior high school, or senior high school), college (2-year college or special training school), and university or higher (university or graduate school). Women were asked how many days per week they worked during early and middle pregnancy, and a response of > 0 days was defined as working. Changes in work status during pregnancy were categorized as not working in early pregnancy, working in early pregnancy only, and working in both early and middle pregnancy.

Women were asked to choose one of the following response options for alcohol use in early and middle pregnancy: current drinker, past drinker, never drinker, and constitutionally never drinker. Alcohol use in the present study was defined as the response “current drinker” based on the idea that there is no known safe amount of alcohol to drink during pregnancy [2, 4]. Alcohol cessation between early and middle pregnancy was defined as alcohol use in early pregnancy, but not in middle pregnancy.

As covariates, we chose age and income [7]. Age in early pregnancy was categorized into three groups: ≤29, 30–34, and ≥ 35 years. Women were asked to select their total annual household income among seven categories: < 2, 2–4, 4–6, 6–8, 8–10, 10–12, and > 12 million Japanese yen. Equivalent household income was calculated as the household income divided by the square root of the number of family members [26], and categorized into four groups: ≤1.99, 2.00–2.99, 3.00–3.99, and ≥ 4.00 million Japanese yen, which nearly corresponded to quartiles.

### Statistical analysis

Multivariable log-binomial regression analyses were conducted to examine the associations of education and work status with alcohol use in early pregnancy, alcohol use in middle pregnancy, and alcohol cessation between early and middle pregnancy. We examined the interactions between education and work status and detected the significant interactions for all three outcomes: *P* = 0.023 for alcohol use in early pregnancy, *P* = 0.019 for alcohol use in middle pregnancy, and *P* = 0.010 for alcohol cessation between early and middle pregnancy. Based on these results, we examined the associations of education with alcohol use and alcohol cessation by work status and the associations of work status with alcohol use and alcohol cessation by education. The prevalence ratios (PRs) and 95% confidence intervals (CIs) were calculated adjusted for age and income.

All analyses were conducted with SAS version 9.4 software (SAS Institute Inc., Cary, NC). For all analyses, a two-tailed *P* value < 0.05 was considered statistically significant.

## Results

### Characteristics of participants

Table 1 shows the characteristics of the pregnant women. About 30% of women had graduated from university or higher. The prevalence of working in early and middle pregnancy was 66.2 and 61.5%, respectively. The prevalence of alcohol use in early and middle pregnancy was 20.9 and 6.4%, respectively.

**Table 1:** Characteristics of the pregnant women: the Tohoku Medical Megabank Project Birth and Three-Generation Cohort Study (*n* = 11,839)

	n	(%)
Educational attainment		
High school or lower	3751	(31.7)
College	4602	(38.9)
University or higher	3486	(29.4)
Work status in early pregnancy		
Not working	4002	(33.8)
Working	7837	(66.2)
Work status in middle pregnancy		
Not working	4555	(38.5)
Working	7284	(61.5)
Alcohol use in early pregnancy		
No	9359	(79.1)
Yes	2480	(20.9)
Alcohol use in middle pregnancy		
No	11,078	(93.6)
Yes	761	(6.4)
Age in early pregnancy		
≤ 29 years	3886	(32.8)
30–34 years	4517	(38.2)
≥ 35 years	3436	(29.0)
Equivalent household income (/year)		
≤ 1.99 million Japanese yen	2692	(22.8)
2.00–2.99 million Japanese yen	3746	(31.6)
3.00–3.99 million Japanese yen	2229	(18.8)
≥ 4.00 million Japanese yen	3172	(26.8)

### Associations of education and work status with alcohol use in early pregnancy

Table 2 presents the prevalences, PRs, and 95% CIs for alcohol use in early pregnancy. Higher educational attainment was associated with an increased probability of alcohol use in early pregnancy regardless of work status; the adjusted PRs of university education or higher compared with high school education or lower were 1.62 (95% CI, 1.34–1.96) among non-working women and 1.29 (95% CI, 1.16–1.45) among working women. Working in early pregnancy was associated with an increased probability of alcohol use in early pregnancy regardless of education; the adjusted PRs of working compared with not working were 1.57 (95% CI, 1.33–1.85) among women with high school education or lower, 1.48 (95% CI, 1.30–1.69) among women with college education, and 1.19 (95% CI, 1.03–1.38) among women with university education or higher.

**Table 2:** Associations of education and work status with alcohol use in early pregnancy according to work status/education

	Alcohol use in early pregnancy/participants	(%)	Crude			Adjusted^a^		
			PR (95% CI)		*P*-value	PR (95% CI)		*P*-value
Educational attainment								
Not working in early pregnancy (*n* = 4002)								
High school or lower	178/1509	(11.8)	1.00			1.00		
College	243/1510	(16.1)	1.36	(1.14–1.63)	< 0.001	1.28	(1.07–1.54)	0.008
University or higher	205/983	(20.9)	1.77	(1.47–2.13)	< 0.001	1.62	(1.34–1.96)	< 0.001
Working in early pregnancy (*n* = 7837)								
High school or lower	422/2242	(18.8)	1.00			1.00		
College	767/3092	(24.8)	1.32	(1.19–1.46)	< 0.001	1.26	(1.13–1.40)	< 0.001
University or higher	665/2503	(26.6)	1.41	(1.27–1.57)	< 0.001	1.29	(1.16–1.45)	< 0.001
Work status in early pregnancy								
High school or lower (*n* = 3751)								
Not working	178/1509	(11.8)	1.00			1.00		
Working	422/2242	(18.8)	1.60	(1.36–1.88)	< 0.001	1.57	(1.33–1.85)	< 0.001
College (*n* = 4602)								
Not working	243/1510	(16.1)	1.00			1.00		
Working	767/3092	(24.8)	1.54	(1.35–1.76)	< 0.001	1.48	(1.30–1.69)	< 0.001
University or higher (*n* = 3486)								
Not working	205/983	(20.9)	1.00			1.00		
Working	665/2503	(26.6)	1.27	(1.11–1.46)	< 0.001	1.19	(1.03–1.38)	0.016

*CI* confidence interval, *PR* prevalence ratio

^a^Adjusted for age and equivalent household income

### Associations of education and work status with alcohol use in middle pregnancy

Table 3 presents the prevalences, PRs, and 95% CIs for alcohol use in middle pregnancy. Educational attainment was not associated with alcohol use in middle pregnancy regardless of work status; the adjusted PRs of university education or higher compared with high school education or lower were 1.23 (95% CI, 0.89–1.71) among non-working women and 0.89 (95% CI, 0.71–1.13) among working women. Working was associated with an increased probability of alcohol use in middle pregnancy among women with high school education or lower and among women with college education; the adjusted PRs of working compared with not working were 1.73 (95% CI, 1.32–2.26) and 1.31 (95% CI, 1.04–1.66), respectively. Working was not associated with alcohol use in middle pregnancy among women with university education or higher; the corresponding PR was 1.05 (95% CI, 0.78–1.40).

**Table 3:** Associations of education and work status with alcohol use in middle pregnancy according to work status/education

	Alcohol use in middle pregnancy/participants	(%)	Crude			Adjusted^a^		
			PR (95% CI)		*P*-value	PR (95% CI)		*P*-value
Educational attainment								
Not working in middle pregnancy (*n* = 4555)								
High school or lower	75/1700	(4.4)	1.00			1.00		
College	98/1720	(5.7)	1.29	(0.96–1.73)	0.087	1.21	(0.90–1.63)	0.213
University or higher	68/1135	(6.0)	1.36	(0.99–1.87)	0.061	1.23	(0.89–1.71)	0.216
Working in middle pregnancy (*n* = 7284)								
High school or lower	154/2051	(7.5)	1.00			1.00		
College	211/2882	(7.3)	0.98	(0.80–1.19)	0.804	0.99	(0.81–1.22)	0.932
University or higher	155/2351	(6.6)	0.88	(0.71–1.09)	0.236	0.89	(0.71–1.13)	0.336
Work status in middle pregnancy								
High school or lower (*n* = 3751)								
Not working	75/1700	(4.4)	1.00			1.00		
Working	154/2051	(7.5)	1.70	(1.30–2.23)	< 0.001	1.73	(1.32–2.26)	< 0.001
College (*n* = 4602)								
Not working	98/1720	(5.7)	1.00			1.00		
Working	211/2882	(7.3)	1.29	(1.02–1.62)	0.034	1.31	(1.04–1.66)	0.024
University or higher (*n* = 3486)								
Not working	68/1135	(6.0)	1.00			1.00		
Working	155/2351	(6.6)	1.10	(0.83–1.45)	0.497	1.05	(0.78–1.40)	0.757

*CI* confidence interval, *PR* prevalence ratio

^a^Adjusted for age and equivalent household income

### Associations of education and work status with alcohol cessation between early and middle pregnancy

Table 4 presents the prevalences, PRs, and 95% CIs for alcohol cessation between early and middle pregnancy among women who consumed alcohol in early pregnancy. The prevalence of alcohol cessation was 77.6%. Higher educational attainment was associated with an increased probability of alcohol cessation among women who worked in early and middle pregnancy; the adjusted PR of university education or higher compared with high school education or lower was 1.09 (95% CI, 1.01–1.17). Education was not associated with alcohol cessation among non-working women and among women who worked only in early pregnancy; the corresponding PRs were 0.95 (95% CI, 0.84–1.08) and 1.09 (95% CI, 0.86–1.38), respectively. Working in early and middle pregnancy was associated with a decreased probability of alcohol cessation among women with high school education or lower but with an increased probability of alcohol cessation among women with university education or higher; the adjusted PRs of working compared with not working in early pregnancy were 0.91 (95% CI, 0.82–1.00) and 1.10 (95% CI, 1.00–1.20), respectively.

**Table 4:** Associations of education and work status with alcohol cessation between early and middle pregnancy according to work status/education among women who consumed alcohol in early pregnancy

	Alcohol cessation/drinkers in early pregnancy	(%)	Crude			Adjusted^a^		
			PR (95% CI)		*P*-value	PR (95% CI)		*P*-value
Educational attainment								
Not working in early pregnancy (*n* = 626)								
High school or lower	139/178	(78.1)	1.00			1.00		
College	182/243	(74.9)	0.96	(0.86–1.07)	0.443	0.96	(0.86–1.08)	0.498
University or higher	151/205	(73.7)	0.94	(0.84–1.06)	0.311	0.95	(0.84–1.08)	0.452
Working only in early pregnancy (*n* = 137)								
High school or lower	33/43	(76.7)	1.00			1.00		
College	54/61	(88.5)	1.15	(0.96–1.39)	0.136	1.19	(0.94–1.51)	0.157
University or higher	29/33	(87.9)	1.15	(0.93–1.41)	0.201	1.09	(0.86–1.38)	0.478
Working in early and middle pregnancy (*n* = 1717)								
High school or lower	273/379	(72.0)	1.00			1.00		
College	553/706	(78.3)	1.09	(1.01–1.17)	0.026	1.06	(0.98–1.14)	0.126
University or higher	510/632	(80.7)	1.12	(1.04–1.21)	0.002	1.09	(1.01–1.17)	0.030
Changes in work status during pregnancy								
High school or lower (*n* = 600)								
Not working in early pregnancy	139/178	(78.1)	1.00			1.00		
Working only in early pregnancy	33/43	(76.7)	0.98	(0.82–1.18)	0.852	0.94	(0.79–1.12)	0.492
Working in early and middle pregnancy	273/379	(72.0)	0.92	(0.83–1.02)	0.113	0.91	(0.82–1.00)	0.048
College (*n* = 1010)								
Not working in early pregnancy	182/243	(74.9)	1.00			1.00		
Working only in early pregnancy	54/61	(88.5)	1.18	(1.05–1.33)	0.005	1.20	(1.05–1.38)	0.010
Working in early and middle pregnancy	553/706	(78.3)	1.05	(0.96–1.14)	0.287	1.04	(0.96–1.12)	0.378
University or higher (*n* = 870)								
Not working in early pregnancy	151/205	(73.7)	1.00			1.00		
Working only in early pregnancy	29/33	(87.9)	1.19	(1.03–1.39)	0.022	1.13	(0.97–1.32)	0.112
Working in early and middle pregnancy	510/632	(80.7)	1.10	(1.00–1.20)	0.048	1.10	(1.00–1.20)	0.048

*CI* confidence interval, *PR* prevalence ratio

^a^Adjusted for age and equivalent household income

## Discussion

The present study examined the associations of education and work status with alcohol use in early and middle pregnancy and alcohol cessation between early and middle pregnancy in Japan. The prevalence of alcohol use decreased from 20.9% in early pregnancy to 6.4% in middle pregnancy. Women with higher education were more likely to consume alcohol in early pregnancy, but were more likely to cease between early and middle pregnancy, especially working women. Working women were more likely to consume alcohol in early and middle pregnancy. Work status was differentially associated with alcohol cessation across educational groups; working women with lower education were less likely to cease alcohol use, whereas working women with higher education were more likely to cease alcohol use.

The prevalence of alcohol use in early pregnancy was 20.9%. The 2013 national survey among the general population of Japan, who may be non-pregnant or pregnant, revealed that women who reported alcohol use comprised 79.4% in their twenties, 77.0% in their thirties, and 77.9% in their forties [23]. Previous studies in Japan showed that about half of pregnant women retrospectively reported alcohol use before they became aware of their pregnancy [9, 10, 21]. Taken together, it is assumed that many women in the present study had already ceased drinking when they filled out the questionnaires administered in early pregnancy.

Higher education was associated with an increased risk of alcohol use in early pregnancy. There are several possible explanations for the association between higher education and alcohol use in early pregnancy. First, alcohol use may be more acceptable among women with higher education. Specifically, more years spent in education, improved labor market prospects, increased opportunities for socialization, and delayed pregnancy mean that alcohol use has easily found a place among these women [27]. Second, social networks among highly educated people may increase the risk of alcohol use. Alcohol use can follow social networking paths [28], and highly educated women tend to associate with other highly educated people [29] who are more likely to consume alcohol [30]. Third, highly educated women may have better-paid jobs involving higher degrees of responsibility and stress as well as more chances to go out drinking with male colleagues with higher limits of drinking [31].

Higher education was also associated with alcohol cessation between early and middle pregnancy among working women. There is some evidence that highly educated women were more likely to consume alcohol before becoming aware of their pregnancy and more likely to continue or reduce rather than cease alcohol use during pregnancy in Western countries [16, 17, 32, 33]. Meanwhile, two studies in Japan showed that highly educated women were less likely to consume alcohol after becoming aware of their pregnancy [9, 21]. Our finding is consistent with the previous findings in Japan. In Japan, it is mandatory for women to notify the municipal office of their pregnancy as soon as it is confirmed. At the municipal office, they receive maternal and child health handbooks [19] and tickets to use for pregnant woman health checkups at public expense. They also have access to counseling services with public health nurses, mother/parent classes, and various information services [20]. Knowledge on the health risks of alcohol use for the fetus was shown to be associated with a decreased risk of alcohol use during pregnancy [10]. Psychological and educational interventions such as supportive counselling and brief educational sessions were suggested to encourage pregnant women to cease alcohol use [34]. It is possible that highly educated women are more receptive to messages offered during the above opportunities than less educated women, because education conveys factual health-related knowledge and raises cognitive skills that affect health-promoting decisions [29, 35]. However, early pregnancy is the time of great neurological vulnerability for the fetus [2, 36]. The message that alcohol can damage a fetus even during the earliest weeks of pregnancy should be spread more widely.

In the present study, working was associated with alcohol use in early and middle pregnancy. The associations between work status and alcohol use during pregnancy are inconsistent [7]; some studies showed that working women had a higher risk of alcohol use during pregnancy than non-working women [9, 37], whereas other studies found no association [38, 39]. One possible explanation is that working may increase the opportunity for alcohol use. In Japan, there is a relatively wide acceptance of alcohol use, and drinking is an important event in some working environments. For example, some working people socialize with colleagues while drinking after work [22, 23]. These working environments may partially explain the observed association between working and alcohol use during pregnancy. The present study also revealed educational interactions with the effect of work status on alcohol cessation during pregnancy; working was associated with a decreased probability of alcohol cessation among women with lower education, while working was associated with an increased probability of alcohol cessation among women with higher education. To the best of our knowledge, this is the first study to demonstrate the interaction between education and work status for alcohol cessation during pregnancy, while socioeconomic interactions with the effect of working on alcohol use have been suggested among women in the general population [40, 41]. Our findings would be helpful in elucidating the complex role of working in alcohol use among women.

Preconception abstinence from alcohol is preferred but difficult, because a large proportion of women of reproductive age consume alcohol [23] and women do not always plan to get pregnant [42]. It is therefore recommended that pregnant women who have already consumed alcohol during pregnancy should stop to minimize further risk [4]. A national campaign in Japan, the second term of Healthy Parents and Children 21, aims to eradicate alcohol use among pregnant women [43]. However, the present study showed that pregnant women consumed alcohol after becoming aware of their pregnancy: 20.9% in early pregnancy and 6.4% in middle pregnancy. For example, the World Health Organization recommends screening all pregnant women about their alcohol use as early as possible in the pregnancy, and offering a brief intervention to all pregnant women who use alcohol [44]. The present study has revealed factors that can be used to identify high-risk subpopulations of pregnant women and targeted in future alcohol-prevention interventions.

The present study has several limitations. First, we were able to analyze approximately half of the pregnant women who agreed to participate in the TMM BirThree Cohort Study. Women who were excluded from the analysis were less educated and more likely to be non-drinkers (Supplementary Table 1), which could lead to underestimation of the association between education and alcohol use during pregnancy. Second, the study was conducted in one of the 47 prefectures in Japan, and the generalizability of the present findings is therefore limited. A national survey reported that the prevalences of alcohol use during pregnancy were 4.3% in 2013, 1.6% in 2015, 1.3% in 2016, and 1.2% in 2017, although these were retrospective reports from mothers after childbirth [43]. Furthermore, there is little information on alcohol use according to pregnancy stage in Japan. Finally, alcohol use was self-reported; this can be a source of uncertainty because women may be influenced by social desirability, a bias that tends to be important when questions deal with socially undesirable attitudes and behaviors. However, the superiority of self-administered questionnaires over face-to-face interviews in measuring alcohol use during pregnancy has been suggested [8].

## Conclusions

Women with higher education were more likely to consume alcohol in early pregnancy and to cease between early and middle pregnancy, especially working women. Working women were more likely to consume alcohol throughout pregnancy, whereas those with lower education were less likely and those with higher education were more likely to cease alcohol use than the corresponding non-working women. Alcohol use during pregnancy is a completely preventable cause of birth defects and developmental disabilities. Our findings indicate that determination of social predictors for alcohol use at different points during pregnancy will be useful for public health interventions to prevent alcohol use among pregnant women.

## Supplementary Information

**Additional file 1 **: **Supplementary Table 1**. Differences in characteristics between 11,839 pregnant women who were analyzed and 9040 pregnant women who were excluded from the analysis.

## Data Availability

Data obtained through the TMM BirThree Cohort Study are incorporated into the TMM biobank. All data analyzed during the present study are available for research purpose with the approval by the Sample and Data Access Committee of the TMM biobank.

## Abbreviations

95% CI: 95% confidence interval
PR: Prevalence ratio
TMM BirThree Cohort Study: Tohoku Medical Megabank Project Birth and Three-Generation Cohort Study
