# Supplementary material for: Associations of education and work status with alcohol use and cessation among pregnant women in Japan: the Tohoku Medical Megabank Project Birth and Three-Generation Cohort Study
Source: BMC Public Health. 2021 Jul 15;21:1400. doi: 10.1186/s12889-021-11461-w (PMC8281686; doi:10.1186/s12889-021-11461-w)
Supplement: Supplementary file 1 — Additional file 1 : Supplementary Table 1. Differences in characteristics between 11,839 pregnant women who were analyzed and 9040 pregnant women who were excluded from the analysis. [file 12889_2021_11461_MOESM1_ESM.pdf]

Supplementary Material

Supplementary Table 1. Differences in characteristics between 11,839 pregnant women who were analyzed and 9040 pregnant women who were excluded from the analysis.

	Women who were analyzed (n=11,839)		Women who were not analyzed (n=9040)		*P*-value[a]
	n	(%)	n	(%)	
Educational attainment					<0.001
High school or lower	3751	(31.7)	636	(41.8)	
College	4602	(38.9)	569	(37.4)	
University or higher	3486	(29.4)	316	(20.8)	
Work status in early pregnancy					0.883
Not working	4002	(33.8)	2817	(33.7)	
Working	7837	(66.2)	5541	(66.3)	
Work status in middle pregnancy					0.559
Not working	4555	(38.5)	3014	(38.9)	
Working	7284	(61.5)	4736	(61.1)	
Alcohol use in early pregnancy					<0.001
No	9359	(79.1)	7337	(81.8)	
Yes	2480	(20.9)	1635	(18.2)	
Alcohol use in middle pregnancy					0.031
No	11078	(93.6)	8435	(94.3)	
Yes	761	(6.4)	510	(5.7)	
Age in early pregnancy					<0.001
≤29 years	3886	(32.8)	3584	(39.6)	
30–34 years	4517	(38.2)	3124	(34.6)	
≥35 years	3436	(29.0)	2332	(25.8)	
Equivalent household income (/year)					<0.001
≤1.99 million Japanese yen	2692	(22.8)	2234	(28.5)	
2.00–2.99 million Japanese yen	3746	(31.6)	2552	(32.5)	
3.00–3.99 million Japanese yen	2229	(18.8)	1360	(17.3)	
≥4.00 million Japanese yen	3172	(26.8)	1699	(21.7)	

Percentages are shown after excluding women whose values were missing.
[a]Obtained using the chi-squared test, comparing women who were analyzed and women who were not.
